# Fine Mapping for Weaver Syndrome in Brown Swiss Cattle and the Identification of 41 Concordant Mutations across NRCAM, PNPLA8 and CTTNBP2

**DOI:** 10.1371/journal.pone.0059251

**Published:** 2013-03-20

**Authors:** Matthew McClure, Euisoo Kim, Derek Bickhart, Daniel Null, Tabatha Cooper, John Cole, George Wiggans, Paolo Ajmone-Marsan, Licia Colli, Enrico Santus, George E. Liu, Steve Schroeder, Lakshmi Matukumalli, Curt Van Tassell, Tad Sonstegard

**Affiliations:** 1 USDA, ARS, ANRI, Bovine Functional Genomics Laboratory, Beltsville, Maryland, United States of America; 2 USDA, ARS, ANRI, Animal Improvement Programs Laboratory, Beltsville, Maryland, United States of America; 3 Istituto di Zootecnica e BioDNA Centro di Ricerca sulla Biodiversità e il DNA Antico, Università Cattolica del S. Cuore di Piacenza, Piacenza, Italy; 4 Associazione Nazionale Allevatori bovini della Razza Bruna, Italian Brown Swiss Association, Bussolengo, Italy; University of Queensland, Australia

## Abstract

Bovine Progressive Degenerative Myeloencephalopathy (Weaver Syndrome) is a recessive neurological disease that has been observed in the Brown Swiss cattle breed since the 1970’s in North America and Europe. Bilateral hind leg weakness and ataxia appear in afflicted animals at 6 to 18 months of age, and slowly progresses to total loss of hind limb control by 3 to 4 years of age. While Weaver has previously been mapped to *Bos taurus* autosome (BTA) 4∶46–56 Mb and a diagnostic test based on the 6 microsatellite (MS) markers is commercially available, neither the causative gene nor mutation has been identified; therefore misdiagnosis can occur due to recombination between the diagnostic MS markers and the causative mutation. Analysis of 34,980 BTA 4 SNPs genotypes derived from the Illumina BovineHD assay for 20 Brown Swiss Weaver carriers and 49 homozygous normal bulls refined the Weaver locus to 48–53 Mb. Genotyping of 153 SNPs, identified from whole genome sequencing of 10 normal and 10 carrier animals, across a validation set of 841 animals resulted in the identification of 41 diagnostic SNPs that were concordant with the disease. Except for one intergenic SNP all are associated with genes expressed in nervous tissues: 37 distal to *NRCAM*, one non-synonymous (serine to asparagine) in *PNPLA8,* one synonymous and one non-synonymous (lysine to glutamic acid) in *CTTNBP2*. Haplotype and imputation analyses of 7,458 Brown Swiss animals with Illumina BovineSNP50 data and the 41 diagnostic SNPs resulted in the identification of only one haplotype concordant with the Weaver phenotype. Use of this haplotype and the diagnostic SNPs more accurately identifies Weaver carriers in both Brown Swiss purebred and influenced herds.

## Introduction

Bovine progressive degenerative myeloencephalopathy (Weaver Syndrome) is a neurodegenerative recessive genetic disorder that has been reported in Brown Swiss pure and crossbred cattle in the USA [1], Switzerland [2], Canada [3], Italy [4], Germany [5], and Denmark [6]. Initial symptoms of progressive hind limb weakness, ataxia, and dysmetria appear in homozygous individuals at 6–18 months of age [7], [8]. While the speed of disease progression varies among cases, the animal’s hind limbs become progressively weaker over the next 2–3 years until it becomes recumbent and is humanely euthanized or dies from malnutrition or infection [6], [8]. Symptoms are caused from degeneration of nerve passages in the spinal cord and brain which prevent the transfer of nerve impulses from the brain to the leg muscles, this degeneration is comparable to Amyotrophic Lateral Sclerosis (ALS) in humans [8], [9]. Occasionally, degeneration and reduction of Purkinje cells occurs in the cerebellum of Weaver affected cattle [6], [10], [11]. While the disease is wholly undesirable, Weaver carriers historically have an economic advantage over non-carriers due to trends in increased milk production [10], [11], [12]. Unfortunately, a number of prominent bulls, notably Rolling View Modern Stretch, were extensively used in the USA before it was discovered they were Weaver carriers [13].

Weaver Syndrome was mapped to *Bos taurus* autosome (BTA) 4 by Georges et al. [10], and the microsatellite (MS) marker *TGLA116* (58.21 Mbp) was identified as a diagnostic marker due to its close linkage (estimated 3% recombination rate) with the locus containing the Weaver allele. The locus was later refined to a 10 Mbp window between markers *BMS2646* and *MAF50* (46.31 and 56.42 Mbp, respectively) [14]. Weaver carrier animals were either identified from affected progeny or via a commercially available genetic test based upon the haplotype of 6 MS markers:RM188, MAF50, RM067, TGLA116, BM1224, and BM6458, within a 43.8 cM region (Ingolf Russ, GeneControl, Germany, personal communication 12/4/2012). Carrier status from the commercial MS test was reported when the confidence level meet or exceeded 90%, but it was not informative for every affected lineage [8].

Cases of Weaver Syndrome affected cattle presumably have been occurring since the 1950’s [15], but the disease was not officially reported in the USA until 1973 [1]. Due to selection for increased milk production and the initial lack of a genetic based diagnostic test, the Weaver allele frequency increased during the late 20^th^ century to an approximate peak of 6% in USA and 5% in Austrian Brown Swiss herds. While the MS test did help identify carrier bulls before their use as breeding stock, the Weaver allele is still present in modern herds with an allele frequency of 2.6% in USA and 3.5% in Austria [16], [17]. While the last confirmed case of Weaver Syndrome in the US was over 10 years ago (Dan Gilbert, Brown Swiss Association, USA (BSUSA), personal communication 11/4/2012), these allele frequencies could be underestimated as affected cattle could go unreported due to a misdiagnosis of arthritis or back injury or they are harvested as veal calves before symptoms appear. Proper diagnosis of Weaver is most accurate when considering the later stages of the disease [18].

A major concern of Brown Swiss Associations is that the Weaver allele frequency is slowly increasing in some herds and breeds, such as Carora, who have used Brown Swiss genetics to improve milk production [19]. This concern is exacerbated in the USA as few, if any bull dams were ever tested and the MS test is not currently used due to a perceived decrease in effectiveness (Dan Gilbert and Dave Kendall, BSUSA, personal communication 5/10/2012). The theory behind reduced effectiveness is that known potential carriers are now multiple generations removed from those being tested and over the years recombination between the Weaver allele and the diagnostic MS has occurred, thus increasing the potential for false negative and positive testing. The potential exists for a large influx of affected individuals if the allele frequency keeps increasing or a non-identified carrier bull becomes widely used. To address these concerns, identify an improved SNP-based diagnostic haplotype, and ascertain potential causative mutation(s), we devised this study to help prevent a sudden increase in Weaver carriers in Brown Swiss purebred and influenced herds worldwide.

## Materials and Methods

### Animals and Phenotypes

A testing population was assembled from cryopreserved semen from 70 Brown Swiss sires and hair roots on 3 dams obtained from the Cooperative Dairy DNA Repository (CDDR) and from the BSUSA. Genomic DNA was isolated by proteinase K digestion followed by Phenol:Chloroform:Isoamyl alcohol extraction, and ethanol precipitation [20] or by using Qiagen miniprep column (Qiagen Sciences Inc, Germantown, MD). DNA concentrations were determined using a Nanodrop 1000 (Thermo Scientific, Wilmington, DE, USA). As all samples were obtained from commercial semen vendors, the CDDR, or hair roots collected and submitted by producers for parentage verification no ethical approval was required to use the samples for this study.

According to BSUSA, 52 of the animals are normal and 21 are Weaver carriers. The disease status of 6 normal and 2 carrier animals was determined via a commercial MS test. The remaining 22 carrier animals were determined via progeny testing (Table S1). At the time of the testing population assembly DNA from an affected Weaver animal, alive or dead, was not available. Unfortunately, DNA and tissue from USA studies in earlier decades were lost due to accidental disposal (David Steffen, personal communication, 6/29/2011).

### HDSNP Genotypes

Seventy-one animals from the testing population (51 normal, 20 carrier) were genotyped using the BovineHD assay (Illumina Inc., San Diego, CA), which interrogates 777,962 evenly spaced SNPs [21] by multiple commercial labs. Genotypes on these animals were obtained from USDA-ARS Animal Improvement Programs Laboratory (AIPL) where they were included in the August 2010, USDA Brown Swiss Genomic Evaluation [22].

Genotypes were filtered for SNP call rate ≥0.90 and autosomal placement on the UMD3.1 assembly [23]. After filtering, 70 animals and 733,937 SNPs remained for analysis, of which 34,980 are located on BTA 4. As the sample call rate for all animals on BTA 4 SNP was >0.98, and the Weaver locus is located on BTA 4 [10], no animals were removed due to call rate filtering.

### Genome-Wide Association Analysis (GWAS)

A GWAS was performed using the testing population (N = 71) and filtered BovineHD SNP using reported Weaver phenotypes with SVS (SNP & Variation Suite) v7.6.2 (Golden Helix, Bozeman, MT). As the testing population contained no affected animals, the data was analyzed as a case/control association test of Weaver carrier versus normal individuals. Pedigree information was included in the analysis. P values were converted to –log10(P_nominal_).

### Familial ALS Gene Associations

Associations of genes in the Weaver locus genes implicated with familial ALS were identified using STRING 9.0 [24]. As no bovine model of ALS exists, *Homo sapiens* protein associations were used. Associations meeting the medium confidence level as defined by STRING were noted.

### Whole Genome Sequencing and Sequence Alignments

Paired-end libraries for 2 Brown Swiss pools with 300 bp inserts were created according to Illumina’s protocol. Pool 1 contained 8 sires and 2 dams that were normal and pool 2 contained 10 sires that were progeny confirmed Weaver carriers, all animals were from the initial testing population (Table S1). To ensure equal sequencing coverage, 1 ug of DNA from each animal was used to create the pool.

Libraries from both pools were sequenced as 2×100 bp paired end libraries on a HiSeq2000 (Illumina), 6 lanes were used for the normal pool and 5 lanes for the Weaver pool. The Weaver library was also sequenced as a 80 bp single read using a GAIIx (Illumina). Sequences were aligned to the UMD3.1 assembly using the Burrows-Wheeler Alignment (BWA) [25], with the Genome Analysis Tool Kit (GATK) realigner used for local realignment around indels and SNP clusters [26]. GATK was run twice, first for indel target determination and then to properly align indels to the reference genome. This decreased the creation of false positive SNP caused by misalignment of sequence data from indels.

Sequence data from SNP Discovery Animals for the Illumina HDSNP assay development (data unpublished) representing 8 breeds (Angus, Brahman, Gir, Holstein, Jersey, Limousine, Nelore, and Romagnola) were also aligned using BWA and GATK for BTA 4.

### Sequence Variation Filtering and Annotation

Sequence variations identified within the refined Weaver locus identified by the GWAS analysis (BTA 4, 48–53 Mb), were filtered for minor allele frequency (MAF) ≥ 0.30 in the Weaver pool and≤0.20 in the Normal pool, and≥6× coverage in the Weaver pool. Stricter filtering was not used to allow for sequencing and alignment errors and to account for potential mis-phenotyped animals in the normal pool. Alleles that were observed in any of the SNP discovery breeds were removed as were ones located in repeat regions (animalgenome.org/repository/cattle/). The annotation of the 153 remaining Weaver candidate variations, all SNP, were estimated using SnpEff version 1.0301 [27].

### Validation Population Assembly and HDSNP Genotyping

A verification population (N = 742) was assembled with 2 confirmed Weaver cases, 26 progeny confirmed carriers, 66 MS test identified carriers, 29 MS test identified normal, and 573 assumed normal animals. Fifty-four animals from the testing population with available DNA were included in the verification population. This population was further increased with 4 Angus, 26 Carora, 4 Holstein, 4 Jersey, 3 Hereford, and 3 Senepol animals derived from other research projects at the Bovine Functional Genomics Laboratory (BFGL). While the verification population is mainly comprised of animals of US descent, 208 Brown Swiss were of Italian descent including the confirmed affected animals which were provided by the Associazione Nazionale Allevatori bovini della Razza Bruna (ANARB). Weaver phenotype status was provided by BSUSA and ANARB.

Weaver affected animals were diagnosed by Italian veterinarians and confirmed via the MS diagnostic test. Animal BS0158 was born 2/15/1999 and diagnosed at 130 days of age, while BS0083 was born 11/16/1999 and diagnosed at 110 days of age. Both had symptoms of weaving walk because of poor control, particularly of rear limbs, and problems in standing up.

All animals’ DNA was obtained via extraction of cryopreserved semen or hair roots using either the method described above, PrepSEQ Nucleic Acid Extraction Kit (Life Technologies, Grand Island, NY), Genomix DNA Extraction Kit (Talent s.r.l, Trieste, Italy), or Chelex 100 [28]. Italian DNA samples were processed by Laboratorio Genetica e Selezione-Associazione Italiana Allevatori (Cremona, Italy) using the above methods. DNA concentrations were determined using a Nanodrop 1000 (Thermo Scientific, Wilmington, DE, USA). As all samples were obtained from commercial semen vendors, the CDDR, hair roots collected and submitted by producers for parentage verification, or certified veterinarians for clinical diagnosis no ethical approval was required to use the samples for this study.

The Weaver affected animal (BS0158), 4 Holstein, 4 Jersey, and 3 Senepol animals were separately HDSNP genotyped at USDA-ARS-BFGL. The second Weaver affected animal (BS0083) had <300 ng of DNA available, therefore it was not HDSNP genotyped. Samples were processed according to the manufacturer’s recommendations and were scanned using a BeadStation 500GX (Illumina) with high-density upgrade. Genotypes were called using BeadStudio v.3 software (Illumina) and filtered for SNP call rate ≥0.90 and autosomal placement on the UMD3.1 assembly.

### Copy Number Variation (CNV) Identification

CNVs were detected using PennCNV as previously described [29]. The dataset was comprised of all Brown Swiss (1 Weaver affected, 19 Weaver carriers, and 32 normal) where HDSNP allele intensity data was available from BFGL or commercial labs. The Hidden Markov Model (HMM) algorithm used to detect CNVs was calibrated by using the expected frequencies of B alleles derived from a previous study [30]. “Genomic waves” in the SNP probe intensity data were normalized by supplying PennCNV with GC% estimates ( (G bases+C bases) / (total bases – assembly gap bases) ) for 1 Mb regions on the UMD3.1 assembly surrounding each probe. CNV detection was limited to the autosomes. All SNP array data passed initial quality filtering steps in the pipeline.

### Targeted SNP Genotyping and GWAS

Multiplex genotyping assays for the 153 Weaver candidate SNPs were designed for the MassARRAY analyzer (Sequenome, San Diego, CA, USA) and genotypes from the verification population at GeneSeek (Lincoln, NE, USA) were loaded into SVS. Illumina BovineSNP50 [31] genotypes were obtained from AIPL for 530 validation animals and from ANARB for 27 Italian animals. ANARB also provided HDSNP genotypes for a 20 Mb region centered on the Weaver locus for 3 Italian animals. A second case/control GWAS was performed with this latter population, using the reported Weaver phenotype as the dependent variable and both Weaver affected and carrier animals analyzed as cases. As not all animals were genotyped for all SNP platforms (Table S1), SNPs were filtered on BTA 4 location, SNP platform call rate ≥0.90, and animal call rate ≥0.80.

### Transcription Factor Binding Site (TFBS) Prediction

Two algorithms were used to detect candidate TFBSs within the Weaver locus. The first, MatInspector [32], was used to detect putative sites using experimentally determined TFBS motifs from the Transfac database [33]. The primary nucleotide sequence for the Weaver locus was used for detection against all vertebrate and core Transfac motifs. Filtering criteria were set to the following values: the core similarity score was set to greater than 0.90 and the matrix similarity score was “opt+0.10” with all other options remaining at the default settings. The core similarity filter only applies to the most conserved nucleotides of the TFBS motif, with higher values indicating better agreement with the consensus sequence. The matrix similarity score represents the conservation of the candidate region against all nucleotides in the TFBS consensus sequence and the MatInspector webserver has optimized detection thresholds for each individual Transfac motif (designated as the “opt” or “optimal” threshold). Since MatInspector does not utilize sequence homology, stringent filtering criteria (0.90 and opt+0.10) were adopted to select only for sequences that had high agreement with experimentally validated TFBS motifs from the Transfac database. These values ensure that the core nucleotides of the motif and the entirety of the matrix itself are represented in each candidate TFBS predicted in the locus. The second algorithm used was aphylogenetic footprinting method adapted from a previous study [34]). Specific details on the methods used and the entire dataset of TFBS predictions can be found in Bickhart and Liu [35]. Since the TFBS predictions in this method were made on the Btau4.0 reference assembly, the UCSC genome browser tool, liftover (http://genome.ucsc.edu/util.html), was used to map the predicted TFBSs to the UMD3.1 assembly.

### Targeted SNP Analysis

An initial genotype frequency analysis of the targeted SNPs revealed 4 Italian Brown Swiss female animals (BS0118, BS0220, BS0217, and BS0129) who were homozygous for the same allele as the known Weaver affected animals for >99% of the targeted SNPs. Further analysis on these 4 animals revealed that they were killed at 6, 15, 17, and 26 months of age, respectively, and that all had a progeny confirmed Weaver carrier ancestor on both their maternal and paternal lineage in≤4 generations. Based on these observations, consistent with non-reported Weaver affected animals, they were not considered for further analysis.

The Carora breed was formed in the 1930’s by using USA and European Brown Swiss semen on Venezuela Criollo cattle [36] With the first cases of Weaver occurring in the 1950’s [7] and an average generation interval of 5–6 years it is highly likely that the Weaver founder animal was born in the 1930’s or earlier. Therefore, it is possible that Weaver carrier Brown Swiss semen was used in the formation of the Carora breed. Allele frequencies in Carora were not used for filtering as this may result in being too conservative or liberal.

The genotypes of the 153 candidate Weaver SNPs were analyzed for their segregation pattern and allele frequency within the different phenotypic groups. The ‘Weaver’ allele for each SNP was determined according to which allele was homozygous in the 2 Weaver affected animals. SNPs were filtered on the following criteria: 1) heterozygous in the affected animals, 2) homozygous in progeny confirmed Weaver carrier animals, 3) homozygous Weaver allele in non affected animals, and 4) Weaver allele frequency (WAF) ≥0.06 (historic WAF highpoint, [16]) in non-affected and non-progeny confirmed carrier animals. The remaining 41 filtered SNPs were considered as diagnostic SNP for later analysis. The filtered SNPs were also checked for a WAF of 0.00 in non-Brown Swiss animals.

### 50K Plus Target SNPs Weaver Haplotype in Brown Swiss

Brown Swiss animals from the validation population (N = 582) who had Weaver candidate SNPs and 50K genotypes in the AIPL Brown Swiss North American (BSNA) database were used to determine Weaver haplotype. Animals were filtered on call rate≥0.9 for the targeted SNPs. The resulting 573 animals had their filtered targeted SNPs (N = 141) added to their 50 k genotypes. The BSNA database also contained 6,362 additional animals with 50K genotypes, 468 with GoldenGate Bovine3K [37], 170 BovineLD [38], 224 GeneSeek Genomic Profiler [39], and 234 dams with imputed genotypes.

Haplotypes were obtained with version 2 of the Fortran program findhap.f90 [40], [41]. The program output haplotypes with a maximum length of 150 markers, covering 2.1 Mb, for further analysis (Table S2). The Weaver haplotype was identified by looking for the most common haplotype in the Weaver locus among animals designated as carriers. Crossover haplotypes were identified through findhap.f90 or by having at least a 50% overlap with the original haplotype [17]. Crossover haplotypes were identified as carrier haplotypes if they shared the same SNPs in the Weaver region as the original haplotype.

### Holstein Breed Composition and Haplotype Prevalence in Breed

One of the Holstein negative control animals (HO1) was homozygous for 32 concurrent Weaver diagnostic SNPs (Table S3). The composition HO1 was estimated using breed-specific markers for Holstein, Jersey, and Brown Swiss obtained from the animal’s 50K genotype. Approximately 672 SNPs, split evenly among the three evaluated breeds, are used to predict breed composition on the 50K chip [42]. HO1’s estimated breed purity was 96% (4.0% non-Holstein influence). This SNP test may underestimate the actual percentage of other breed’s genes present in the animal.

Next it was determined which animals have the highest relationship with HO1 to trace its non-Holstein ancestry. The percent conflict method, similar to parentage validation, was used to calculate the percentage of opposite homozygous SNP genotypes between HO1 and all genotyped animals [43]. Animals with a lower percentage of opposite homozygous SNP are considered to be more related. From this test, HO1’s ancestry could be traced back to both Ayrshire and Brown Swiss animals. Of the 25 most-related Brown Swiss bulls to this Holstein animal, five are progeny confirmed Weaver carriers.

HO1 was commercially used for artificial insemination and has 718 recorded progeny. To investigate the prevalence of the Holstein haplotype from this bull containing the SNP haplotype for Weaver, all genotyped Holsteins in the AIPL database were imputed using methods by VanRaden et al. [41] to a common set of 45,187 markers. The genotypes were then broken into 75-marker haplotype blocks and the haplotype frequency was calculated based upon animals that contained an identical haplotype or crossover haplotype to HO1 [17].

## Results

### GWAS, TFBS, and CNV

**Figure 1:**
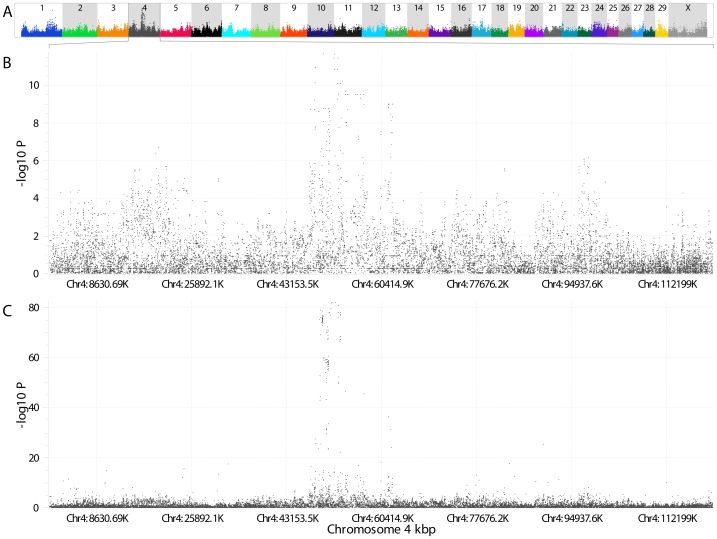
Weaver Syndrome GWAS. GWAS results using reported Weaver phenotypes for 72 individuals with HDSNP genotypes for **A**) all BTAs **B**) BTA 4. **C**) GWAS results for BTA 4 using reported Weaver phenotypes for 829 individuals with target, 50K, and/or HD genotypes. UMD3.1 coordinates used.

GWAS analyses on both the testing and validation populations resulted in a reduction of the Weaver locus from 46–56 Mb to 48–53 Mb on BTA 4 (Figure 1). Multiple CNV (N = 2,961) were identified across the genome in Weaver carrier animals with 99 located on BTA 4. No CNV were located in the refined 5 Mb Weaver locus, but 2,243 unique TFBS were.

### Sequence Variations

Whole genome sequencing of the two Brown Swiss pools and alignment to the UMD3.1 assembly resulted in an average of 21X coverage for pool 1 and 22X for pool 2 across the Weaver locus as redefined by the GWAS results. Within the locus 50,814 variations were identified, of which there were 4,570 insertions, 4,137 deletions, and 42,107 SNPs. These were filtered down to 153 SNPs unique to Brown Swiss and most were determined to be intergenic by annotation analysis (Table S4). Four SNPs failed to produce any genotypes and 15 were monogenic in the validation population leaving 134 SNPs for analysis (Table 1, Table S3). Of the remaining SNPs, a high concentration, 30%, were located between the annotated gene boundaries of *NRCAM* and *PNPLA8* (49,651,802–49,867,702 bp).

**Table 1:** Target SNP interactions and filter information.

										Filtering results							
			Diagnostic SNP interaction							Weaver Allele							
Position	SNP	Weaver allele^a^	TFBS^b^		Bovine EST^c^		Non-bovine mRNA		dbSNP^d^	Not homozyougs in affected	Homozygous in non-affected	Frequency >0.06 in non-affected	Frequency >0.00 in control breeds	Homozygous in all animals	Homozygous in progeny confirmed carriers	SNP call rate <0.90	No genotype produced
48007166	A/G	G														X	
48007166	A/G	–															X
48232691	G/A	–								X		X	X				
48337968	G/A	–								X							
48402338	C/A	C										X		X			
48486736	C/T	–								X		X	X				
48512459	T/C	C										X		X			
48615216	T/C	–								X		X	X				
48622306	C/A	–								X		X	X				
48732707	T/A	–								X		X	X				
48735699	T/C	–								X		X	X				
48901850	C/T	–								X		X	X				
48947509	T/C	T														X	
48955770	C/T	T										X				X	
49037435	G/A	A										X		X			
49114986	C/T	C										X			X	X	
49138874	A/G	A									X						
49244789	G/A	G									X	X	X				
49247979	T/G	T									X	X	X		X		
49258127	T/C	T									X	X	X				
49290010	T/G	T									X						
49310747	A/C	C									X	X	X		X		
49323692	T/C	T									X	X					
49331946	C/T	C									X				X		
49388225	A/C	A									X	X	X		X		
49389293	T/C	T									X	X	X		X		
49403179	C/G	C									X	X	X		X		
49526344	C/T	C									X						
49651768	G/A	G															
49653001	T/C	T									X						
49656945	T/C	T					X^e^		rs109982331								
49657798	G/A	A			X												
49664852	T/C	T	X														
49667361	T/C	T							rs109664911								
49673503	T/C	T							rs109895716				X^g^				
49681169	T/C	C											X^g^				
49682552	G/A	G	X						rs110232598				X^g^				
49686038	G/A	G											X^g^				
49687224	G/T	G											X^g^				
49687585	T/C	T							rs110652080				X^g^				
49691015	G/T	T	X						rs109108372				X^g^				
49691127	T/C	C										X		X			
49692015	C/T	C											X^g^				
49692485	T/G	T							rs108969425				X^g^				
49692825	T/C	T							rs110864204				X^g^				
49693121	T/C	C											X^g^				
49693140	G/T	T											X^g^				
49693164	T/A	T											X^g^				
49693178	C/A	C											X^g^				
49693265	G/A	G											X^g^				
49693284	G/A	A											X^g^				
49693601	T/C	C											X^g^				
49693913	C/A	C											X^g^				
49694977	G/A	G											X^g^				
49695504	G/A	A							rs110503389				X^g^				
49695952	T/C	T							rs110633416				X^g^				
49698002	T/C	T							rs109252615				X^g^				
49698436	T/C	T											X^g^				
49700154	G/A	A											X^g^				
49701106	T/C	C											X^g^				
49702287	T/C	C							rs109980500				X^g^				
49702494	G/A	A											X^g^				
49704844	G/A	G									X		X^g^				
49705951	G/A	G	X										X^g^				
49706022	T/C	C					X^f^						X^g^				
49708283	T/C	C											X^g^				
49715678	T/C	T	X						rs109748527				X^g^				
49718641	T/C	T							rs109194289				X^g^				
49850942	G/A	A														X	
49878773	T/C	T															
49906971	G/T	G									X	X					
49959480	A/G	A									X	X					
49976213	G/T	G									X	X					
50223808	T/C	C									X	X	X				
50274978	G/A	G									X	X					
50298949	C/T	C									X	X					
50312908	G/A	G									X	X					
50333552	T/C	T									X	X					
50360030	A/C	A									X	X					
50366763	A/G	–								X		X	X				
50367956	T/A	T									X	X					
50369238	G/A	A										X				X	
50379409	A/G	G														X	
50379409	A/G	–															X
50380664	G/T	T										X				X	
50393360	T/C	T									X	X			X		
50398187	G/A	A									X	X			X		
50398280	T/C	T									X	X			X		
50402642	G/C	G									X	X			X		
50455808	T/C	T									X	X			X		
50472537	G/A	A										X					
50475447	G/A	G										X					
50481151	G/A	G															
50534843	G/T	G									X	X					
50541402	T/C	T										X					
50543512	T/C	T									X	X					
50552996	G/A	G									X	X					
50553258	G/A	G									X	X					
50554161	T/C	C									X	X					
50557292	G/A	A									X	X					
50557504	T/C	T									X	X					
50561765	G/A	A									X	X					
50564926	T/A	T									X	X					
50567814	T/C	T									X	X			X		
50608260	A/G	–								X		X					
50634196	T/C	C							rs111022650							X	
50634196	C/T	–															X
50644398	G/A	A										X					
50675770	T/C	C										X					
50705601	A/C	A									X		X				
50705673	C/A	C									X	X	X				
50728982	T/A	T									X	X	X				
50741937	G/A	G									X	X	X		X		
50752507	C/T	C									X	X					
50755178	A/G	A									X	X					
50757722	A/G	A										X					
50758316	G/A	G									X	X					
50759242	T/C	T									X	X					
50769426	G/T	G									X	X					
50771856	T/C	C										X				X	
50772507	A/G	A									X	X					
50772537	A/G	A									X	X					
50774184	T/G	T														X	
50774778	T/C	T									X	X					
50774884	C/T	C									X	X					
50775126	C/T	C									X	X					
50779324	G/A	G									X	X	X				
50790384	A/G	A									X	X	X		X		
50796591	A/G	A									X	X					
50858538	A/G	A															
50929556	G/A	G															
51077321	T/C	T									X						
51096233	G/A	G										X	X			X	
51216265	T/C	T									X		X				
51387621	G/C	C									X						
51395221	G/A	G									X						
51828149	T/C	C										X			X	X	
51836464	G/C	C										X					
52023948	A/G	A									X						
52447753	A/C	C										X		X			
52488585	G/C	G									X						
52561658	T/C	C									X						
52588273	C/T	C														X	
52594374	G/A	G									X	X	X		X		
52614817	T/C	T									X	X	X		X		
52615034	C/A	C									X	X	X		X		
52849464	G/A	G									X	X			X		
52851362	A/G	A									X	X			X		
52853963	T/C	T									X	X			X		
52895919	A/G	A									X	X	X		X		
52900386	T/C	T									X	X			X		
52934522	T/C	T									X	X			X		
52934540	C/T	C									X				X		
52938145	T/G	T										X			X	X	
52967096	A/G	A									X						
52976777	T/G	T									X	X					

a The homozyous allele in affected animals.

b Affected predicted TFBS information in Table 3.

c Bovine EST: AJ677346.

d None of the listed dbSNP IDs are validated.

e mRNA (n = 56) at SNP 49656945, partial list: JO339806, JU559277, JV452054, AK382080, BC024441, AK231430.

f mRNA (N = 4) at SNP 49706022: JO339806, JO397144, AK382080, JO62179.

g SNP is heterozygous in one Holstein bull with 4% non Holstein genomic influence.

Fifteen SNPs were present in dbSNP (Table 1), but the dbSNP assignment is based on one Fleckvieh bull who was resequenced in 2009. dbSNP indicates that none of these variations are validated and because they derive from one animal they potentially represent sequencing errors. Therefore the dbSNP information is noted in Table 1, but the SNPs were not filtered out in our analysis.

### Diagnostic SNPs

Filtering on genotype and allele frequencies in the Weaver affected and non-affected groups left 41 SNPs (Table 1). Thirty-seven of them are grouped together at 49.65–49.72 Mb. Of interest, 49656945 and 49706022 were located in multiple non-bovine aligned mRNA; 49657798 was in an aligned bovine EST; and 49664852, 49682552, 49691015, 49705951, and 49715678 altered the conserved binding sites of predicted TFBS; with the remaining being intergenic (Table 1, Table 2). Of the remaining SNPs, 3 were located in coding regions: 49878773 a non-synonymous in *PNPLA8* (serine to asparagine), 50858538 a synonymous in *CTTNBP2*, and 50929556 a non-synonymous (lysine to glutamic acid) in *CTTNBP2.*

**Table 2:** Predicted TFBS that are affected by targeted Weaver SNP.

		TFBS				Similarity score^b^	
SNP		Start^a^	End	Name	direction	Reference base	Alternative base
49664852	C/T	49664849	49664856	TGACCTTG	+	918	876
49682552	A/G	49682545	49682558	HNF1A	−	776	under threshold^c^
		49682550	49682561	PBX1	+	836	under threshold
		49682551	49682556	Pdx1	−	912	under threshold
		49682550	49682562	Lhx3	+	851	under threshold
		49682547	49682559	Lhx3	−	786	under threshold
		49682551	49682562	Foxa2	−	865	under threshold
		49682551	49682558	HOXA5	+	under threshold	898
		49682552	49682557	ARID3A	+	940	under threshold
		49682552	49682557	ARID3A	−	940	under threshold
		49682545	49682556	HNF1B	−	791	under threshold
		49682550	49682556	YCATTAA	+	865	under threshold
		49682544	49682554	RGTTAMWNATT	+	794	under threshold
		49682552	49682559	TGATTTRY	−	868	under threshold
		49682551	49682558	YATTNATC	+	985	940
		49682545	49682557	RYTAAWNNNTGAY	−	787	under threshold
		49682545	49682555	TAAYNRNNTCC	−	867	under threshold
49691015	T/G	49691005	49691016	RTTTNNNYTGGM	+	842	under threshold
49704844	A/G	49704842	49704851	CCAWWNAAGG	−	783	783
49708283	C/T	49708278	49708291	Evi1	−	782	814
		49708279	49708284	Pdx1	+	930	896
		49708278	49708284	GATAAGR	−	under threshold	850

a UMD3.1, chromosome 4 positions.

b Comparision of TFBS for reference and alternative base pair.

c Predicted TFBS’s PSSM similarity value was below the threshold and was not called for this allele. Often this is due to a change in the consensus binding site of the TFBS by the polymorphic base of the SNP.

Analysis of the diagnostic SNP allele frequencies in the non-Brown Swiss breeds revealed a Holstein (HO1) that was homozygous normal for 5 SNPs, heterozygous for next 32 SNPs between *NRCAM* and *PNPLA8* and then homozygous normal for the remaining 4 SNPs (Table S3). Analysis of HO1 revealed that its genome has up to 4% non-Holstein origin and has distant Brown Swiss and Ayrshire ancestors.

In most cases, an animal heterozygous for the 37 SNPs flanking *NRCAM* was typically heterozygous for the remaining 4 diagnostic SNPs (Table S3). Exceptions to this are BS0310 and BS0146 who have no reported Weaver phenotype (Table S3). Both are heterozygous for the 37 SNPs, but BS0146 is homozygous normal for 49878773, 50858538, and 50929556 while BS0146 is homozygous normal for 50481151, 50858538, and 50929556 (Table S3). BS0146’s sire, BS0185 (a reported Weaver carrier by MS testing) and his paternal half-sibs, BS0114 and BS0117, are part of the validation population and all are homozygous normal for all 41 SNPs. Additionally animal BS0418 is homozygous normal for all of the diagnostic SNPs, except 49878773, for which it is heterozygous (Table S3).

### Identification of Misidentified / Unidentified Carrier Brown Swiss Animals

Based upon the genotypes for the 41 diagnostic SNPs, new phenotypes were assigned to all Brown Swiss with SNP call rates >0.80. A Weaver carrier status was given if the animal was heterozygous and normal status given if homozygous for the non-Weaver allele for all 41 SNPs. Based upon this, 4 animals that were originally reported as carriers from the MS test were called normal, one normal from the MS test was called a carrier, 3 unreported and 1 MS reported carrier were called Weaver affected, 65 unreported were called carrier, and 570 unreported were called normal, with all other animals’ assigned and reported phenotypes matching. Three unreported animals (BS0418, BS0310, and BS0146) were called unknown as they were not fully heterozygous or homozygous for all 41 SNPs. The same assignment was made for the Carora and of them 21 were called normal and 5 carrier (Table S3).

From the haplotype analysis of 7,458 Brown Swiss with 50K or imputed 50K data 563 haplotypes were identified, of which only one, haplotype #2, was identified as the Weaver carrier haplotype (Table S5). Application of the Weaver haplotype identified 257 carrier animals and 7,201 non carriers, with birth years ranging from 1951 to 2011 (data not shown). Assigned phenotype agreed for all animals with a phenotype assigned according to both the diagnostic SNPs and haplotype analysis method.

### Haplotype Prevalence in Holstein

A low (0.03) haplotype frequency (data not shown) was identified in the Holstein breed for animals containing an identical haplotype or crossover to HO1, who was heterozygous for the majority of the diagnostic SNPs. With such a low frequency, the opportunity for a carrier-by-carrier mating is very low, and may explain why the disease has not been documented in the Holstein breed.

## Discussion

The refined Weaver locus (48–53 Mb) contains 17 genes with predicted associations with *SOD1, TDP-43, FUS*, *VCP,* and *PFN1* (Table 3). Mutations causing familial ALS (FALS) have been identified in *SOD1* [*44*], *TDP-43* [*45*], *FUS* [*46*], *VCP* 4 [47], and *PFN1* [*48*]. While FALS is autosomal dominant and Weaver is autosomal recessive, they share multiple phenotypes and similar nerve degeneration patterns [9]. Of the 2,961 CNVs identified across the genome in Weaver carriers none were located in the refined Weaver locus of 48–53 Mb. The apparent lack of CNV in the Weaver locus is also consistent with a FALS comparison, as multiple rare CNVs have been implicated as a potential risk factor for sporadic ALS in humans [49], but not with FALS [50]. The high concentration of ALS associated genes (Table 3) supports the refinement of the Weaver locus by the initial GWAS. While the second GWAS incorporated 785 animals with targeted SNPs plus either 50K or HDSNP genotypes (Table S1), it failed to further refine the interval defined in the first GWAS, but did increase the statistical support sevenfold (Figure 1). Filtering for SNP segregation patterns in the various phenotype groups left 41 diagnostic SNPs that lie in or near 3 genes: *NRCAM* (neuronal cell adhesion molecule), *PNPLA8* (patatin-like phospholipase domain containing 8) and *CTTNBP2* (cortactin binding protein2).

**Table 3:** Predicted associations between genes in Weaver locus and familial ALS.

Gene	SOD1^a^	TDP-43	FUS	VCP	PFN1
PIK3CG	3^b^	4	2	4	4
LOC100140892					
PRKAR2B	2	2	4	**2**	3
LOC100336179					
HBP1					
GPR22					
COG5					
DUS4L					
BCAP29					
SLC26A4					
CBLL1			3		
SLC26A3	2	2	4	3	3
DLD	**1**	3	5	3	3
LAMB1	2	3	3	3	4
MIR2418					
NRCAM	3	4	4	4	5
PNPLA8					
CTTNBP2	4	2	3	2	4
LOC784535					
TXNDC3					
SFRP4	4	3	5	3	3
EPDR1					
STARD3NL					
LOC100336170					
MIR2417					
TRGC6					
LOC530341					
TCRG	3	4	6	4	2
CFTR	3	1	4	**1**	3
ASZ1					
WNT2	3	2	4	2	2
ST7	3	3	5	3	2
LOC100337386					
CAPZA2	**2**	3	5	3	1
LOC100138854					
MET	4	3	2	3	5
LOC781951					
CAV1	5	4	**3**	4	6
LOC100140425					
CAV2	6	5	**3**	5	7
LOC100296613					
TES					

a Associations predicted by STRING (Jensen et al. 2009).

b Count of association steps between genes.

**Figure 2:**
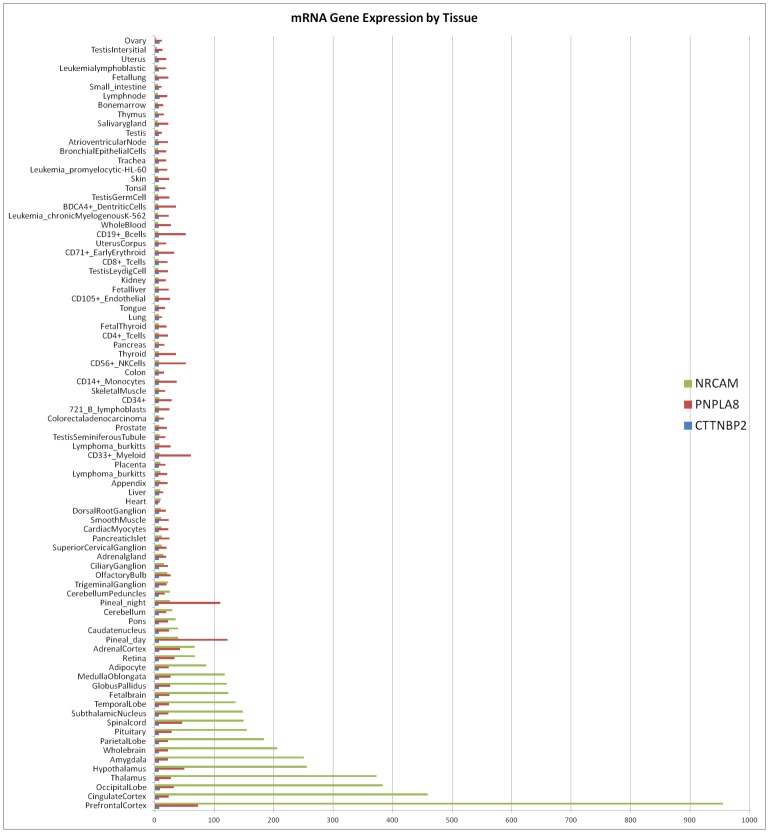
mRNA expression levels of NRCAM, PNPLA8, CTTNBP2. Graph created using data from www.biogps.org (accessed 7/18/2012) for mRNA expression levels of the gene’s mRNA expression across 84 mouse and humans tissues. While few gene expression across multiple tissues have been done in bovine, it is logical to expect a similar expression pattern as shown above given the high gene exon homology of PNPLA8, CTTNBP2, and NRCAM between bovine, mouse, human, and other mammalian species (ENSEMBL).

*NRCAM* encodes for neuronal adhesion molecule, an ankyrin-binding protein that is involved in neuron-neuron adhesion and promotes directional signaling during axonal cone growth (www.uniprot.org). *NRCAM* is engaged in such biological processes as axonal fasciculation, cell-cell adhesion, central nervous system development, clustering of voltage-gated sodium channels, neuron migration, positive regulation of neuron differentiation, regulation of axon extension, and synaptogenesis. It also may play a role in the molecular assembly of the nodes of Ranvier. *NRCAM* is linked with different recognition processes and signal transduction pathways regulating cell differentiation, proliferation, or migration [51], [52]. While expressed in multiple tissue types it is highly expressed in human and mouse nervous tissues (Figure 2) and should have an analogous expression in bovine. It may play a general role in cell-cell communication via signaling from its intracellular domain to the actin cytoskeleton during directional cell migration. *NRCAM* promotes directional signaling during nervous system development in several different regions as the spinal cord, the visual system, and the cerebellum [53], [54]. *NRCAM* is associated with mammalian phenotypes for abnormal axon morphology, locomotor behavior/coordination, motor coordination/ balance, nervous system electrophysiology, neuron morphology, voluntary movement, nerve conduction, neurite morphology, ataxia, paralysis, and reduced nerve conduction velocity (PhenomeNet, accessed 7/19/2012 [55]). NRCAM is expressed in the spinal cord and cerebellum (Figure 2) which are both used for clinical validation of Weaver Syndrome [6], [10], [11].

*PNPLA8* encodes for the enzyme calcium-independent phospholipase A2-gamma and is a member of the patatin-like phospholipase domain containing protein family which catalyzes the cleavage of fatty acids from membrane phospholipids and serve critical roles in transducing cellular signals in to biologically active lipid 2^nd^ messengers [56]. *PNPLA8* has significant upregulation during adipocyte differentiation, is involved in facilitating lipid storage in adipocyte tissue energy mobilization, and maintaining mitochondrial integrity [57], [58]. In mice, *PNPLA8* knockouts show significant motor abnormalities and cognitive deficits over time, associated with synaptic loss and α-synuclein accumulation in brain [59].

*CTTNBP2* encodes for the protein cortactin-binding protein 2 and is expressed exclusively in brain neurons [60]. It regulates dendritic spinogenesis through interaction with cortactin and knockdown of *CTTNBP2* reduces the dendritic spine distribution of cortactin [61]. *CTTNBP2* may direct cortactin-dependent actin dynamics at dendritic spines and control spine morphology and density. The contribution of *CTTNBP2* to dendritic spine formation indicates that *CTTNBP2* might participate in controlling cognitive functions related to autism or other psychiatric disorders [62].

**Figure 3:**
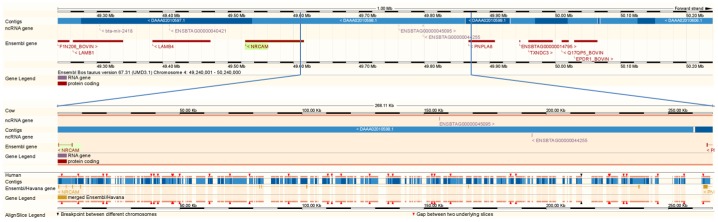
Ensembl alignment of the bovine and human annotations from NRCAM to PNPLA8. Bottom portion of the figure highlights the annotated human exons (brown cross hatch) for NRCAM which align to an unannotated portion of the bovine genome. UMD3.1 assembly used.

The *NRCAM* annotated size between bovine (BTA4∶49,526,574–49,615,802) and human (HSA7∶107,788,082–108,097,161) differs by >219 Kb. Alignment of the 2 genome annotations shows that *NRCAM* has 7 exons, not present in the bovine annotation, which narrows the gap between *NRCAM* (BTA4∶49,526,574–49,615,802) and *PNPLA8* (BTA4∶49,867,702–49,907,595) to <20 Kb based on the human annotation, versus a 252 Kb gap on the bovine annotation (Figure 3). In the unannotated region of the bovine genome that aligns with the extended human annotation for NRCAM there are 9 bovine ESTs (AJ677248, DR115601, DT884253, AJ677346, CO889715, EE239088, EE240414, EE912749, and EE912738), 3 ncRNA (www.ensembl.org, accessed 7/18/2012, [63], and >60 non-bovine mRNA (examples: Capra J0339806, Ctenomy JU559277, Macaca JC452054, Bombyx AK382080, Mus AK082725, and Centris JI000402) (www.genome.ucsc.edu, accessed 7/20/2012). Of the 37 diagnostic SNPs that lie between the bovine annotations of *NRCAM* and *PNPLA8,* one is in bovine EST DR115601, two overlay non-bovine EST that align to the region, and five affect predicted TFBS (Table 1). The extra human *NRCAM* exons, non-bovine mRNA alignments, and predicted TFBS suggest that *NRCAM* is mis-annotated for bovine and potentially other non-human species.

Weaver is a neurological disease and while *NRCAM, PNPLA8,* and *CTTNBP2* are all expressed in nervous tissues, overall *NRCAM* is expressed at higher levels across all nervous tissues, including the spinal cord (Figure 2). This study cannot rule out the possibility that SNPs in *CTTNBP2* (50858538 and 50929556), the intergenic SNP 50481151, or the four SNPs which failed to produce genotypes (48007166, 49850942, 50379409, and 50634196) are the causative mutation for Weaver. But the gene expression patterns, biological processes affected, associated phenotypes, and density of target SNP strongly suggest that one of the 37 SNPs distal to *NRCAM* or the non-synonymous SNP in *PNPLA8* (49878773), affects the gene in a manner that ultimately results in Weaver Syndrome in homozygous animals.

A low (0.03) haplotype frequency was identified the Holstein breed for animals containing an identical haplotype or crossover to HO1, who was heterozygous for the majority of the diagnostic SNPs. If HO1 is a Weaver carrier, then the low haplotype frequency and therefore very low random opportunity for a carrier-by-carrier mating may explain why the disease has not been documented in the Holstein breed. If HO1 is a non-carrier, then this haplotype represents a small level of Brown Swiss introgression in Holstein and also further reduces the Weaver SNP haplotype. HO1 was born in 1976 and has had 718 registered progeny with the youngest born in 2008. Unfortunately none of his offspring have SNP genotypes in the AIPL database.

### Conclusion

While a single conclusive causal allele was not identified in this study we were able to identify a reduced list of potential causative SNPs. The combined analysis of targeted SNP genotypes and imputed haplotypes resulted in the identification of 285 new Brown Swiss and 5 Carora Weaver carriers. Selective breeding of animals like BS0310, BS0146, and BS0418 who are heterozygous for some of the 41 diagnostic SNPs and homozygous for others to known carrier animals would allow for a further reduction of potential diagnostic SNP based upon which homozygous SNP are present in affected animals. While use of the diagnostic SNPs and haplotypes reported here will be beneficial to identify Weaver carriers in both Brown Swiss pure and crossbred animals, continuing research needs to take place to identify the true causative Weaver mutation.

Regardless of which SNP is the causative mutation, the imputation of the diagnostic SNPs for animals with 50K genotypes and their haplotype analysis resulted in >7,500 animals having their Weaver phenotype determined. At a modest $30 cost of using a commercial lab to extract DNA and genotype the Weaver diagnostic SNPs this represents a combined $225,000 cost saving for the owners of these animals. An added benefit is that this diagnostic SNP imputation and haplotype analysis can be performed on future animals with low-density SNP genotypes that can be imputed accurately up to 50K, until genotyping platforms are able to economically add the diagnostic SNP reported here or the causative allele(s) is determined by further research.

## Supporting Information

Table S1 Target SNP interactions and filter information.
(XLSX)

Table S2 140 SNP Weaver Haplotype.
(XLSX)

Table S3 Target SNP genotpyes and pedigree information.
(XLSX)

Table S4 Predicted effect of filtered sequence variations.
(XLSX)

Table S5 Weaver 50K+Target SNP Haplotypes.
(XLSX)
